# Roles of Organellar RNA-Binding Proteins in Plant Growth, Development, and Abiotic Stress Responses

**DOI:** 10.3390/ijms21124548

**Published:** 2020-06-26

**Authors:** Kwanuk Lee, Hunseung Kang

**Affiliations:** 1 Plant Molecular Biology (Botany), Department of Biology I, Ludwig-Maximilians-University München, 82152 Martinsried, Germany; 2 Department of Applied Biology and AgriBio Institute of Climate Change Management, Chonnam National University, Gwangju 61186, Korea

**Keywords:** organellar gene expression, chloroplast, mitochondria, RNA metabolism, RNA-binding proteins, abiotic stress

## Abstract

Organellar gene expression (OGE) in chloroplasts and mitochondria is primarily modulated at post-transcriptional levels, including RNA processing, intron splicing, RNA stability, editing, and translational control. Nucleus-encoded Chloroplast or Mitochondrial RNA-Binding Proteins (nCMRBPs) are key regulatory factors that are crucial for the fine-tuned regulation of post-transcriptional RNA metabolism in organelles. Although the functional roles of nCMRBPs have been studied in plants, their cellular and physiological functions remain largely unknown. Nevertheless, existing studies that have characterized the functions of nCMRBP families, such as chloroplast ribosome maturation and splicing domain (CRM) proteins, pentatricopeptide repeat (PPR) proteins, DEAD-Box RNA helicase (DBRH) proteins, and S1-domain containing proteins (SDPs), have begun to shed light on the role of nCMRBPs in plant growth, development, and stress responses. Here, we review the latest research developments regarding the functional roles of organellar RBPs in RNA metabolism during growth, development, and abiotic stress responses in plants.

## 1. Introduction

Plant chloroplasts and mitochondria are thought to be derived from free-living cyanobacteria and α-proteobacteria, respectively [1,2]. During evolution, the organellar genes were largely transferred to the nucleus [2]. Current organellar genomes harbor only 15–209 proteins in the chloroplast, and 3–67 proteins in the mitochondrion [3], which are essential for photosynthetic apparatus, mitochondrial electron transport chain, and organellar gene expression (OGE) machinery [4]. OGE in plant organelles conserves both prokaryotic and eukaryotic properties [5]. However, the OGE mechanisms in plant organelles are much more complex than those of their bacterial ancestors [5,6] and require thousands of nucleus-encoded proteins for maintaining OGE machinery and organellar function. This indicates the importance of interactions between the organelles and nucleus in controlling fine-tuned OGE through a nucleus-to-organelle anterograde or an organelle-to nucleus retrograde signaling [7,8,9]. 

OGE is commonly regulated at the post-transcriptional level, including RNA processing, editing, stabilization, turnover, intron splicing, and translational control, all of which are crucial for a number of organellar processes [10,11,12,13]. The regulation of post-transcriptional RNA processing in organelles requires hundreds of nucleus-encoded chloroplast or mitochondrial RNA-binding proteins (nCMRBPs) during acclimation to environmental stress, as well as during plant growth and development [14,15]. Recent studies have uncovered that nCMRBPs play a critical role in plant growth and stress responses [15,16,17,18,19]. Moreover, analysis of the characteristics of nCMRBP has demonstrated that they possess multiple conserved motifs and domains, which include chloroplast RNA splicing and ribosome maturation (CRM), pentatricopeptide repeat (PPR), DEAD-box RNA helicase (DBRH), and S1 RNA-binding domain (SDP) [19,20,21,22]. Importantly, it is now known that the nCMRBPs function as either specific RNA-binding proteins or non-specific RNA-binding proteins (RNA chaperones), which facilitates the correct folding of the target RNA structure during plant growth and under environmental stress [15,23]. Chloroplast- or mitochondria-localized CRM, PPR, DBRH, and SDP proteins have been assessed in terms of their roles as RNA chaperones [21,24,25,26,27]. In this review, we will focus on the recent advances in research on the function and cellular mechanisms of CRM, PPR, DBRH, and SDP proteins in organellar RNA metabolism during plant growth, development, and abiotic stress responses.

## 2. Domain or Motif Features of CRM, PPR, DBRH, and SDP Proteins

### 2.1. CRM Proteins

**Figure 1:**
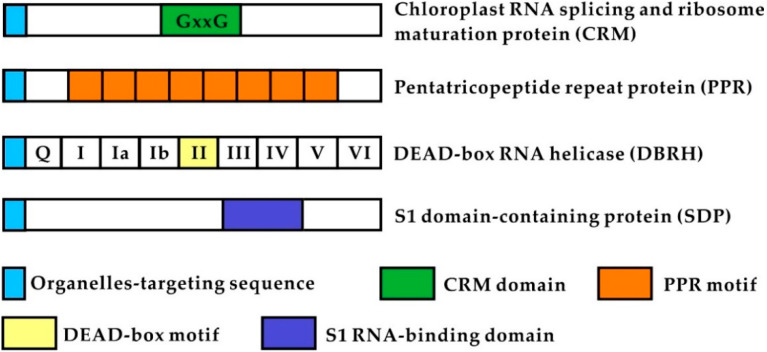
Basic domains or motifs of chloroplast ribosome maturation and splicing domain (CRM), pentatricopeptide repeat (PPR), DEAD-Box RNA helicase (DBRH), and S1-domain containing proteins (SDP) proteins in plants.

Single chloroplast RNA splicing and ribosome maturation (CRM) domain-containing proteins were first studied in archaea and bacteria [28,29], in which domain analysis revealed that CRM is orthologous to *E. coli* YhbY associated with pre-50S ribosomal subunits [30] (Figure 1). Land plants harbor single to multiple copies of CRM domains that can be classified into 4 subfamily groups, which include the CRS1 subfamily (for chloroplast RNA splicing), the CAF subfamily (for CRS2-associated factors), and CFM3 and 4 (for CRM family members), based on the proteome database of *Arabidopsis* and rice [20]. Furthermore, structural analysis has demonstrated that GxxG sequences conserved in the loop of the CRM domain contribute to RNA-binding capacity [20,30,31]. 

### 2.2. PPR Proteins

Pentatricopeptide repeat proteins were first identified in the *Arabidopsis* genome and are comprised of tandem repeated motifs of 35-amino acid sequences, ranging from 2 to 30 tracts [23,32,33]. PPR motifs fold into a pair of antiparallel α helices and contribute to organellar RNA metabolism on the basis of modular one-repeat:one-nucleotide binding [23,34]. Plant PPR proteins are classified into two subfamilies; P- and PLS-class (P: 35 amino acids, L: 36 amino acids, and S: 31 amino acids, Figure 1). Most P-class proteins contain only PPR motifs, although some also harbor a PPR-small MutS-related (SMR) domain, which is important for organellar RNA stabilization, group II intron splicing, and intercistronic processing [23]. In contrast, the PLS-class proteins contain additional C-terminal domains of E, E^+^, and DYW, which are mainly involved in RNA C to U editing via recruiting additional proteins [33,35]. 

### 2.3. DEAD-Box RH Proteins (DBRH)

Helicase proteins are enzymes that catalyze the unwinding of double-stranded DNA or duplex RNA secondary structures in ATP-dependent rearrangements in both prokaryotic and eukaryotic cells [36,37]. They are divided into six superfamilies (SF1–SF6) based on the properties of the conserved motifs in their primary amino acid sequences [38,39]. The DBRH family belongs to the largest group, superfamily 2 (SF2), and harbors at least nine conserved motifs, such as Q, I, Ia, Ib, II, III, IV, V, and VI (Figure 1) [40,41,42]. The Q-motif, motif I (walker A motif), motif III, and motif VI of the DBRH family are essential for ATP binding and ATP hydrolysis [41,43,44]. Motif II (walker B motif), which contains residues of Asp-Glu-Ala-Asp (DEAD) is also crucial for ATP binding and ATP hydrolysis via the interaction of Mg^2+^ [39,41]. Only a few biochemical studies have been focused on the remaining (Ia, Ib, IV, and V) motifs. However, it has been suggested that they are also involved in RNA binding [41]. 

### 2.4. SDP Proteins

The S1 RNA domain-containing protein (SDP) was first observed in the ribosomal proteins S1 (RPS1) of *E. coli*. [45]. *E. coli* RPS1 is comprised of six copies of an S1 motif containing approximately 70 amino acids [46]. The structure of the S1 domain adopt a five-stranded antiparallel β barrel in which residues Phe-19, Phe-22, His-34, Asp-64, and Arg-68 are believed to infer its RNA-binding ability [46]. S1 domain repeats (Figure 1) vary from one to 15 in different species [47] and have been identified in RNase E endonuclease (RNase E), RNase II exonuclease (RNase II), transcription factor NusA, and *C. elegans* EMB-5 [46], which play a crucial role in mRNA turnover, rRNA processing, and translational initiation [48,49,50,51]. In addition, S1 domain repeats are also found in other RNA-associated proteins, such as bacterial polynucleotide phosphorylase (PNPase) [52,53], bacterial translation initiation factor 1 (IF1), eukaryotic eIF2a [54], and the RNA helicase-like protein PRP22 found in yeasts [55]. Furthermore, a recent study has demonstrated that the amino acid sequence homologies of S1 domains are approximately 43% in archaea, 51% in bacteria, and 46% in eukaryotes, and that the residues of Phe-28, Asp-66, and Arg-71 in archaea and Phe-25, Asp-68, and Arg-71 in eukaryotes are highly conserved [47]. These findings suggest that S1 domains are diverse with a low sequence identity among different species.

## 3. Functions of nCMRBPs in Plant Growth and Development

The latest studies have indicated the importance of nCMRBPs, including CRM, PPR, DBRH, and SDP, for organellar RNA metabolism during plant growth and development (Table 1). Fourteen and 16 CRM proteins are encoded in *Arabidopsis* and rice genomes, respectively [16,20]. Previous analysis has indicated that chloroplast-localized *Arabidopsis* AtCRS1 [56], AtCAF1 [56], AtCAF2 [56], AtCFM2 [28], and rice OsCFM3 [57], and dual-localized AtCFM3 [57] in both chloroplasts and mitochondria, are involved in the splicing of subsets of specific introns. The recent functional analysis of unknown CRM subfamilies has uncovered that *Arabidopsis* AtCFM4 [24] is involved in *16S* and *23S rRNA* processing, and that rice OsCAF1 [58] and OsCFM2 [59] is important for the splicing of chloroplast introns as the orthologues of AtCAF1 and AtCAF2. Furthermore, it has been demonstrated that mitochondria-localized *Arabidopsis* mCSF1 [60] and CFM9 [61] are involved in the splicing of multiple mitochondrial introns and can influence seed development and seedling growth, respectively, indicating that CRM proteins play a crucial role in plant growth and development. 

As the structural characteristics of PPR motifs were first determined in plants, the functions of a large number of plant PPR proteins have been reported over the last 20 years [23,26]. As it is not possible to consider all of these in this review, we will only discuss the P-type PPR proteins. Previous analysis of PPR proteins has demonstrated that chloroplast-localized maize PPR4 [62] and THA8 [63], and *Arabidopsis* OTP51 [64] and OTP70 [65], are essential for the splicing of chloroplast specific introns. Interestingly, recent studies of PPR4 [25], EMB2654 [66], PBF2 [67], and SOT5 [68] in *Arabidopsis* showed that these proteins play a role in the splicing of chloroplast introns through their role in the recognition of the specific RNA sequences. *Arabidopsis* HCF152 [69], MRL1 [70], PGR3 [71], BFA2 [72], and maize PPR5 [73] and PPR10 [74] were shown to be essential for the stabilization of chloroplast transcripts. AtPPR2 [75], SOT1 [76], and PPR287 [77] in *Arabidopsis* are involved in chloroplast rRNA processing. In addition, mitochondria-localized *Arabidopsis* OTP43 [78], BIR6 [79], TANG2/OTP439 [80], and SLO3 [81] are known to affect the splicing of mitochondrial introns. The latest studies of *Arabidopsis* MISF 26, 68, 74 [82], and EMB2794 [83] have also demonstrated their significance in mitochondrial intron splicing. Furthermore, *Arabidopsis* MTSF1 [84] and PPR19 [85] were found to be important for mitochondrial RNA stabilization as they bind to specific sequences, suggesting that organelle-localized PPR proteins perform versatile roles in organellar RNA metabolism.

Approximately 58 and 50 DBRH were annotated from the *Arabidopsis* and rice genome, respectively [86,87]. Although the functional roles of DBRH in plants have been investigated for several decades, the DBRH functions in the chloroplast and mitochondria are not as well understood as those in the nucleus. Nonetheless, the roles of DBRHs for organellar RNA metabolism have been emerged [26,88,89,90,91]. In *Arabidopsis,* chloroplast-localized RH3 [26] and ISE2 [92] and mitochondria-localized PMH2 [93] are involved in the splicing of diverse organellar introns, and RH22 [94], RH39 [95], and RH50 [89] are associated with chloroplast rRNA processing and ribosome biogenesis. In addition to this, chloroplast-localized SDP proteins, including *Arabidopsis* RLSB [96] and *Nicotiana* STF [97], play roles in plastid gene expression, supporting the notion that *Arabidopsis* SDP is crucial for chloroplast rRNA processing during plant growth and development. 

Importantly, the aforementioned nCMRBPs are transported into chloroplasts and/or mitochondria, and mutations in these genes result in various phenotypes, including embryo lethality, albino, pale green, dwarfism, delayed growth, as well as impaired photosynthesis and mitochondrial respiration (Table 1). This indicates that nCMRBPs play central roles in a variety of cellular RNA metabolism processes in organelles during plant growth and development.

**Table 1:** Phenotypes and functions of CRM, PPR, DBRH, and SDP proteins in plant growth and development.

Plant	Gene Name	Gene Number	Location	Molecular Function	Mutant Phenotype	Ref.
*A. thaliana*	CRM family					
	*AtCRS1*	At5g16180	C	Splicing of group II intron (*atpF*)	Small and albino seedling	[28,56]
	*AtCAF1*	At2g20020	C	Splicing of group II introns (*petD, rpl16, rps16, ndhA, rpoC1, ycf3-1, clpP-1,* and *trnG*)	Albino seedling	[56]
	*AtCAF2*	At1g23400	C	Splicing of group II introns (*ndhA, ndhB, petB, ycf3-1,* and *rps12-1*)	Small and pale green seedling	[56]
	*AtCFM2*	At3g01370	C	Splicing of group I (*trnL*) and group II introns (*ndhA, ycf3-1,* and *clpP-2*)	Small and albino seedling	[28]
	*AtCFM3a*	At3g23070	C/M	Splicing of group II intron (*ndhB*)	Stunted growth	[57]
	*CFM4*	At4g39040	C	*16S* and *23S rRNA* processing	Retarded growth	[24]
	*mCSF1*	At4g31010	M	Splicing of multiple mitochondrial introns	Embryo lethal Retarded growth	[60]
	*CFM9*	At3g27550	M	Splicing of multiple mitochondrial introns	Retarded growth	[61]
*O. sativa*	*OsCAF1*	Os01g 0495900	C	Splicing of group II introns (*atpF, rpl2, rps12, ndhA, ndhB,* and *ycf3*)	Albino seedling	[58]
	*OsCFM2*	Os04g 0464800	C	Splicing of group I (*trnL*) and group II introns (*atpF, rpl2, rps12, ndhA,* and *ycf3-1*)	Albino seedling	[59]
	*OsCFM3*	Os11g 37990	C	Splicing of group II introns (*ndhB, petD, rpl16, rps16, trnG*, and *petB*)	Albino seedling	[57]
*A. thaliana*	PPR family					
	*OTP51*	At2g15820	C	Splicing of *ycf3* intron2	Pale yellow seedling	[64]
	*OTP70*	At4g25270	C	Splicing of *rpoC1* intron	Virescent seedling	[65]
	*AtPPR4*	At5g04810	C	*Trans*-splicing of *rps12* intron1	Embryo lethal, pale green, or albino seedling	[25]
	*EMB2654*	At2g41720	C	*Trans*-splicing of *rps12* intron1	Embryo lethal, pale green, or albino seedling	[66]
	*PBF2*	At3g42630	C	Splicing of *ycf3* intron1	Small and pale yellowish seedling	[67]
	*SOT5*/*EMB2279*	At1g30610	C	Splicing of *rpl2* and *trnK* intron	Virescent seedling	[68]
	*HCF152*	At3g09660	C	Stabilization or processing of *psbB-psbT-psbH-petB-petD*	High chlorophyll fluorescence	[69]
	*MRL1*	At4g34830	C	Stabilization of *rbcL*	Pale green seedling	[70]
	*PGR3*	At4g31850	C	Stabilization of *petL* and probably *ndhA*	High chlorophyll fluorescence	[71]
	*BFA2*	At4g30825	C	Stabilization of *atpH/F*	Stunted growth	[72]
	*AtPPR2*	At3g06430	C	Chloroplast *23S rRNA* processing	Embryo lethal or albino seedling	[75]
	*SOT1*	At5g46580	C	Chloroplast *23S-4.5 rRNA* processing	Small and pale green seedling	[76]
	*PPR287*	At4g59040	C	Processing of chloroplast *16S, 23S, 4.5S,* and *5S rRNAs*	Yellowish seedling	[77]
	*OTP43*	At1g74900	M	*Trans*-splicing of *nad1* intron1	Small and delayed development	[78]
	*BIR6*	At3g48250	M	Splicing of *nad7* intron1	Small and retarded growth	[79]
	*TANG2* *OTP439*	At1g19290 At3g48810	M	Splicing of *nad5* intron2 and 3	Retarded growth	[80]
	*SLO3*	At3g61360	M	Splicing of *nad7* intron2	Delayed growth and development	[81]
	*MISF26* *MISF68* *MISF74*	At1g66345 At3g16010 At4g01400	M	Splicing of *nad2* intron3 (MISF26) Splicing of *nad2* intron2, *nad4* intron1, and *nad5* intron4 (MISF68) Splicing of *nad1* intron4 and *nad2* intron4 (MISF74)	Delayed growth	[82]
	*EMB2794*	At2g02150	M	*Trans*-splicing of *nad2* intron2	Retarded growth and developmental defect	[83]
	*MTSF1*	At1g06710	M	Stabilization of *nad4*	Retarded growth	[84]
	*PPR19*	At1g52620	M	Stabilization of *nad1* intron3	Retarded growth and developmental defect	[85]
*Z. mays*	*PPR4*	Zm00001d026654	C	*Trans*-splicing of *rps12* intron1	Seedling lethal pale green, or albino seedling	[62]
	*THA8*	GRMZM2G466032	C	Splicing of *ycf3* intron2 and *trnA* intron	Pale green seedling	[63]
	*ZmPPR5*	GRMZM2G025409	C	Splicing of *trnG* intron	Seedling lethal or pale green seedling	[73]
	*PPR10*	GRMZM2G177169	C	Stabilization of *atpH* and *psaJ*	Seedling lethal or yellowish green seedling	[74]
*A. thaliana*	DBRH family					
	*RH3*	At5g26742	C	Splicing of group II introns (*trnI, trnA, rps12-1, rps12-2,* and *rpl2*) and chloroplast *23S rRNA* processing	Embryo lethal or pale green seedling	[88]
	*ISE2*	At1g70070	C	Splicing of group II introns (*rpl2, atpF, rps12,* and *clpP*)	Chlorotic seedling	[92]
	*PMH2*	At3g22330	M	Splicing of *nad2* introns	Similar to wild-type	[93]
	*RH22*	At1g59990	C	Chloroplast *23S-4.5S rRNA* processing	Embryo lethal or virescent seedling	[94]
	*RH39*	At4g09730	C	Chloroplast *23S rRNA* processing	Retarded growth	[95]
	*RH50*	At3g06980	C	Chloroplast *23S-4.5S rRNA* maturation	Similar to wild-type	[89]
*A. thaliana*	SDP family					
	*SDP*	At1g12800	C	Processing of chloroplast *16S, 23S, 4.5S,* and *5S rRNAs*	Pale green seedling	[21]
	*RLSB*	At1g71720	C	Regulation of *rbcL* mRNA	Reduced seedling size	[96]
*N. benthamiana*	*STF*	HM012811	C	Regulation of plastid transcription	Yellowish leaves	[97]

## 4. Physiological Functions of nCMRBPs in Abiotic Stress Responses

As sessile organisms, plants often face adverse environmental conditions, including extremes of temperature, high salinity, drought, and UV stresses, all of which can severely damage crop productivity and yield [98,99]. To survive these harsh conditions, plants need to adapt to these environmental challenges by reprogramming the expression of genes in their nucleus, chloroplasts, and mitochondria [18,100,101]. The organelles serve as a stress sensor, and the regulation of OGE [100,102] and organellar metabolic processes are essential for acclimatizing to abiotic stress responses [16,18]. A number of studies have determined the functional roles of nCMRBPs in organelles for environmental stress responses (Table 2). 

Chloroplast-localized *Arabidopsis* CRM-containing CFM4 (*16S* and *23S rRNA* processing) has been determined as a positive effector in seed germination and seedling growth under low temperature and salt stress conditions [24]. Recent work to characterize mitochondria-localized *Arabidopsis* CFM9, which is involved in the splicing of multiple mitochondrial introns, has demonstrated its positive role in seed germination and seedling growth in the presence of the abscisic acid (ABA) and under high salinity or dehydration stress [61]. Although the *cfm4* and the *cfm9* mutants grew slowly under normal conditions, the mutant characteristics of growth retardation and delayed germination were much more severe under abiotic stress conditions compared to those of the wild type. This indicates that organelle-targeted CFM4 and CFM9 also play a crucial role in plant responses to abiotic stresses. 

The diverse roles of organelle-localized PPR proteins have been demonstrated in the responses of plants to abiotic stresses. The loss-of-function mutant of chloroplast-localized *Arabidopsis* GUN1 was found to be hypersensitive to sucrose and ABA [103]. Chloroplast-localized rice WSL, which is involved in the splicing of chloroplast *rpl2* introns, enhanced seed germination and seedling growth in response to multiple environmental factors, such as glucose, ABA, and salinity, owing to its reduced translation efficiency [104]. Chloroplast-localized rice OsV4 affects the gene expression of plastid translation machinery, TCD10 is important for the gene regulation of *OsV4*, *OsRpoTp*, *V1*, *V2*, *RNRL*, *RNRS*, *16S rRNA*, *rpl21,* and *OsDG2*, and WSL5 are involved in the editing of *rpl2* and *atpA,* as well as the splicing of *rpl2* and *rps12* intron2, are crucial for chloroplast biogenesis, the mutants of which lead to albino or pale yellowish phenotypes during cold stress [105,106,107]. The overexpression of mitochondria-localized *Arabidopsis* PPR40 has been shown to promote seed germination in the presence of salt or ABA and improve seedling growth under conditions of high salinity by reducing reactive oxygen species (ROS) damage in the mitochondria [108,109]. In addition, mitochondria-localized *Arabidopsi*s PGN, which is involved in the expression of mitochondrial *NAD1*, *RPL2*, *NAD9*, and *MATR* genes, plays a role in both biotic and abiotic stress tolerance, and its loss-of-function mutants are susceptible to ABA, salt, and glucose, as well as necrotrophic fungal pathogens [110]. *Arabidopsis* ABO5 and ABO8, which are involved in the splicing of mitochondrial *nad2* intron3 and *nad4* intron3, respectively, have been shown to have enhanced sensitivity to ABA during post-germination and root growth phase due to the accumulation of ROS in the mitochondria [111,112]. Interestingly, *Arabidopsis* PPR96, which is thought to be involved in mitochondrial RNA editing, has a negative impact on seed germination and seedling growth [113].

The organelle-localized DBRHs are essential for the responses of plants to environmental stresses. The loss-of-function mutant of chloroplast-localized *Arabidopsis* RH3, which is involved in the splicing of *ndhA* and *ndhB* introns, displays hypersensitivity to salt and cold stress, and to ABA [26]. Recently, cold-inducible rice TCD33, which is thought to be involved in chloroplast ribosome assembly, has been shown to affect chloroplast biogenesis under cold stress [91]. Moreover, the ectopic expression of rice RH58, which is involved in the translation of chloroplast *POR*, *RBCL*, *CLPB3*, *PSBA*, and *PETA* transcripts, and cabbage RH22, which affects the translation of chloroplast *RBCL*, *PSBA*, and *YCF3* genes, contributed to an enhanced tolerance to salt and drought stress in *Arabidopsis* by increasing the translational efficiency of chloroplast mRNAs [90,114].

Chloroplast-localized SRRP1 harboring two S1 domains was shown to decrease sensitivity to ABA by impairing the splicing of the chloroplast *trnL* intron and *5S rRNA* processing in the presence of ABA [115]. Additionally, the overexpression of chloroplast RPS5, which is involved in *16S rRNA* processing, enhanced seedling growth in response to cold stress [116]. A recent study has also demonstrated that chloroplast-localized SDP, which affects rRNA processing in chloroplasts under normal conditions, has positive effects on salt, heat, freezing, or UV stress tolerance, as it influences the stress-responsive genes in the nucleus [117]. Beyond organelles, it will be of great interest to investigate how nCMRBP-mediated organellar retrograde signaling (ORS) influences the reprogramming of the expression of stress-responsive nuclear genes, and the organellar and nuclear epigenetic modifications for stress priming and memory [101,118,119,120,121]. Although the study of organellar proteomics and metabolomics is far behind than those in the nucleus and cytoplasm, recent studies emphasize the importance of homeostasis between the nucleus and organelles in plant acclimation to environmental changes [122,123,124,125]. With these omics data, future tasks are to identify novel ORS molecules and pathways, which will widely expand our understanding of crosstalk between the nucleus and organelles.

**Table 2:** Phenotypes and functions of CRM, PPR, DBRH, and SDP proteins in abiotic stress responses.

Plant	Gene Name	Gene Number	Location	Molecular Function	Mutant Phenotype	Ref.
*A. thaliana*	CRM family					
	*CFM9*	At3g27550	M	Splicing of multiple mitochondrial introns	Sensitive to salt, drought, or ABA	[61]
	*CFM4*	At4g39040	C	*16S* and *23S rRNA* processing	Sensitive to salt or cold stress	[24]
*A. thaliana*	PPR family					
	*ABO5*	At1g51965	M	Splicing of *nad2* intron3	Sensitive to ABA	[111]
	*PPR40*	At3g16890	M		Sensitive to salt, ABA, or oxidative stress Tolerant to salt stress in overexpression plants	[108,109]
	*GUN1*	At2g31400	C		Sensitive to sucrose or ABA	[103]
	*ABO8*	At4g11690	M	Splicing of *nad4* intron3	Sensitive to ABA	[112]
	*PPR96*	At2g03380	M	Probably mitochondrial RNA editing	Tolerant to salt, ABA, or oxidative stress	[113]
	*PGN*	At1g56570	M	Regulation of *NAD1, RPL2, NAD9,* an*d MATR* genes	Sensitive to salt, glucose, or ABA	[110]
*O. sativa*	*OsV4*	Os04g39970	C	Plastid gene expression associated with plastid translation machinery	Sensitive to cold stress	[105]
	*WSL*	Os01g37870	C	Splicing of chloroplast *rpl2* intron	Sensitive to salt, sucrose, or ABA	[104]
	*TCD10*	Os10g28600	C	Regulation of *OsV4, OsRpoTp, V1, V2, RNRL, RNRS, 16S rRNA, rpl21,* and *OsDG2* genes	Sensitive to cold stress	[106]
	*WSL5*	Os04g0684500	C	RNA editing of *rpl2* and *atpA*, and splicing of *rpl2* and *rps12* intron2	Sensitive to cold stress	[107]
*A. thaliana*	DBRH family					
	*RH3*	At5g26742	C	Splicing of *ndhA* and *ndhB* introns	Sensitive to salt or cold stress	[26]
*O. sativa*	*TCD33*	Os03g01830	C	Probably chloroplast ribosome assembly	Sensitive to cold stress	[91]
	*OsRH58*	Os01g73900	C	Translational control of chloroplast *POR*, *rbcL, Clpb3, PsbA,* and *PetA* transcripts	Tolerant to salt or drought stress	[90]
*B. rapa*	*BrRH22*	Bra035413	C	Translational control of chloroplast *rbcL, psbA*, and *ycf3* transcripts	Tolerant to salt or drought stress	[114]
*A. thaliana*	SDP family					
	*SRRP1*	At3g23700	C	Splicing of chloroplast *trnL* intron and *5S rRNA* processing	Sensitive to ABA	[115]
	*RPS5*	At2g33800	C	Chloroplast *16S rRNA* processing	Tolerant to cold stress in overexpression plants	[116]
	*SDP*	At1g12800	C	Processing of chloroplast *16S, 23S, 4.5S,* and *5S rRNAs*	Sensitive to UV, salt, heat, or freezing stress	[117]

## 5. Cellular Roles of nCMRBPs in Organellar RNA Metabolism

The mechanistic role of nCMRBPs in plant growth, development, and abiotic stress responses remains largely unknown. However, recent studies have revealed that nCMRBPs act as RNA chaperones in plant growth and development, as well as in stress adaptation processes.

RNA molecules must adopt correct structures in order to maintain functional RNAs. However, RNA molecules are often misfolded into non-functional secondary or tertiary structures in cells, due to intrinsic thermodynamic and kinetic folding problems [126,127]. As such, either specific RBPs or RNA chaperones are required to ensure correct folding. An RNA chaperone is defined as a non-specific RNA-binding protein that guides the folding of RNA molecules to ensure functionally active states are achieved through structural rearrangement [127,128]. RNA chaperones usually bind to a wide range of RNA species and are characterized as being non-specific [129,130]. Another of their typical features is that they do not require external energy input or ATP, and they generally adopt structurally disordered regions rendering RNA chaperone activity.

Research conducted over a number of decades has demonstrated that RNA chaperones are crucial for diverse cellular processes in prokaryotic and eukaryotic organisms [15]. It has been demonstrated that viral nucleocapsid proteins and *E. coli* Hfq and ProQ are important for stress responses because of their roles as RNA chaperones [131,132]. Studies characterizing multiple DBRHs in bacteria, animals, and yeast have demonstrated that CYT-19, DeaD, SrmBp, RhlE, and Mss116p are associated with the splicing of mitochondrial group I and II introns through their RNA chaperone activity [133,134,135]. In plants, it has also been demonstrated that U11/U12-31K, a minor spliceosomal protein of *Arabidopsis* and rice, is involved in the splicing of U12-type introns as an RNA chaperone, and it is essential for the correct folding of introns during normal growth and development [136,137]. Studies of RNA chaperones have also been expanded to a variety of CSDP, GRP, and RZ proteins in *Arabidopsis*, rice, cabbage (*Brasscia rapa*), and wheat (*Triticum aestivum*) under various environmental conditions [15,138,139,140,141,142,143,144,145,146,147]. In plant organelles, recent findings have illustrated that chloroplast-localized *Arabidopsis* CFM4, RH3, SDP, and SRRP1 have RNA chaperone properties that are crucial for maintaining the structures of the precursor-RNA molecules suitable for splicing or rRNA processing [21,24,115,147]. It has also been demonstrated that chloroplast-localized *Arabidopsis* and rice PPR4, containing both RRM and PPR motifs, possess RNA chaperone activity through its RRM motif, and thereby affect the trans-splicing of *rps12* intron1 [25]. Moreover, chloroplast-localized rice OsRH58 and cabbage BrRH22 were shown to affect the translation of multiple chloroplast mRNAs through their RNA chaperone activities that aid in the structural rearrangement of target mRNAs for subsequent efficient translation control under environmental stresses [90,114]. Mitochondria-localized *Arabidopsis* CFM9 was shown to affect the splicing of multiple mitochondrial introns, and the *cfm9* mutant was found to be sensitive to abiotic stresses [61]. As such, CFM9 is presumably important for mitochondrial intron splicing due to its RNA chaperon function. Taken together, these results clearly indicate that nCMRBPs, which carry out RNA chaperone activities, have significant roles in the regulation of organellar RNA metabolism during plant growth, development, and responses to abiotic stress.

## 6. Conclusions and Future Directions

**Figure 2:**
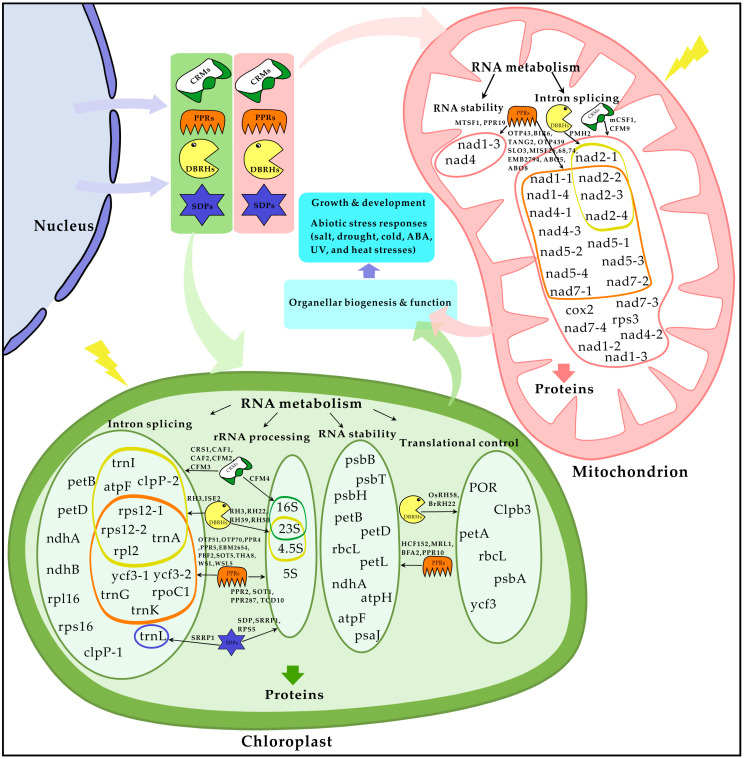
Cellular function of nucleus-encoded Chloroplast or Mitochondrial RNA-Binding Proteins (nCMRBPs) in organellar RNA metabolism. Nucleus-encoded CRMs, PPRs, DBRHs, and SDPs are transported into chloroplasts and/or mitochondria and are involved in RNA metabolism, including intron splicing, RNA stability, rRNA processing, and translational control in organelles as described in Table 1 and Table 2. The nCMRBP-mediated RNA metabolism influences the homeostasis of organellar biogenesis and function, which plays an essential role in plant growth and development, as well as in abiotic stress responses. Yellow-colored thunder indicates environmental stimuli.

Although the functional roles of nucleus-encoded organellar RBPs are still not fully understood, the latest studies of the cellular and physiological functions of nCMRBPs has shed some light on the significance of nCMRBPs for organellar RNA metabolism during plant growth, development, and environmental stress responses. It has been demonstrated that chloroplasts- or mitochondria-localized CRM, PPR, DBRH, and SDP proteins play pivotal roles in organellar post-transcriptional RNA metabolism, including intron splicing, rRNA processing, and translational control under normal and stressful conditions (Figure 2). Since different nCMRBPs often target same RNA for processing or splicing, it would be interesting to determine whether nCMRBPs interact together to mediate RNA metabolism. Moreover, given that the target organellar RNAs of many nCMRBPs are not known yet, determination of the sequence- and structure-dependent recognition of target RNAs by RBPs would be important for further understanding of the mechanistic roles of nCMRBPs. In particular, many nCMRBPs have been shown to play their roles as RNA chaperones that aid in the structural rearrangement of RNA molecules during plant growth and responses to environmental stimuli. However, further research is required to unravel the mechanisms underlying the RNA chaperone function and to identify any protein partners that may interact with nCMRBPs, which play indispensable roles in organellar RNA metabolism under both normal conditions and abiotic stress. In addition to this sequence- and structure-dependent RNA regulation, epitranscriptomic RNA methylation is recently emerging as a new form of post-transcriptional RNA regulation associated with plant development and stress responses [148,149,150]. However, to date, the significance of RNA methylation in the recognition of target RNAs by RBPs, and the importance of the interactions between RBPs and modified RNAs, have yet to be determined. With the recent advances in high-throughput methylated RNA immuneprecipitation-sequencing technology [151], transcriptome-wide m^6^A methylation patterns in the chloroplast and mitochondria RNAs have been reported [152]. It would be interesting to determine how the methylation in organellar RNAs influences the recognition and subsequent binding of nCMRBPs to target RNA. This knowledge will further our understanding of the regulation of RNA metabolism in organelles that are essential for stress adaptation, as well as plant growth and development.
